# Impact of gender on post- traumatic intensive care and outcomes

**DOI:** 10.1186/s13049-019-0693-4

**Published:** 2019-12-23

**Authors:** Emma Larsson, Ann-Charlotte Lindström, Mikael Eriksson, Anders Oldner

**Affiliations:** 1 Perioperative Medicine and Intensive Care, Karolinska University Hospital, Solna, SE-171 76 Stockholm, Sweden; 2 Section of Anaesthesiology and Intensive Care Medicine, Department of Physiology and Pharmacology, Karolinska Institutet, Stockholm, Sweden

**Keywords:** Trauma. Gender. Intensive care

## Abstract

**Background:**

Several reports indicate gender disparities in health care provision. There is a well-documented male patient dominance in intensive care unit (ICU) admittance. It is not established if this difference reflects medical needs or is influenced by other factors. The aim of the current study was to investigate if patient gender influences the pattern of ICU admittance in a cohort of trauma patients.

**Methods:**

Data from patients admitted to an urban trauma centre over a 10-year interval were linked to regional and national health registries to obtain data on demographics, co-comorbidities, trauma-related variables, ICU-admittance patterns and mortality. The association between gender and ICU-admission were explored using logistic regression analysis. The association between gender and short- and long-term mortality were explored using Cox regression models.

**Results:**

In this study cohort of approximately 14,000 trauma patients, men had a higher probability of being admitted to the ICU after initial trauma resuscitation. The difference was limited to patients with less severe injuries (ISS < 15). No differences were noted in short-term survival, whereas men had a higher long-term mortality.

**Conclusions:**

In this retrospective cohort study we found a difference between men and women in post trauma ICU admittance patterns, restricted to less injured patients, where men had a higher probability of ICU admittance. Whether this is a true gender bias or an effect of other factors not analysed in this study remains unknown. This finding warrants further studies.

## Introduction

There are several reports regarding gender disparities in various fields of health care. Among patients treated in intensive care units (ICU) there is a well-known male dominance where men constitute approximately 60% [1–3]. Given the assumption that treatment in the ICU is beneficial for critically ill patients, one could speculate if this difference may imply a survival advantage for men. Up to date, such data has not been reported. Aside from differences in ICU admission rates it has also been reported that men consume more ICU resources while admitted [1–3]. As ICU resources often are limited, correct utilization is of paramount importance. The male dominance among trauma patients could be a plausible explanation of the gender differences in trauma related ICU populations. Differences in mortality after trauma between men and women has also been a subject of debate, but it is hard to draw any clear conclusions from previous studies [4–7]. Gender disparities in trauma patients and associated ICU care are scarcely investigated and several topics within this field remain to be explored. The aim of the current study was to investigate if patient gender influences the pattern of ICU admittance in a cohort of trauma patients. We hypothesized that male trauma patients are admitted to the ICU to a higher extent than their female counterparts, with comparable injury severity. Secondary aims were to investigate short- and long-term mortality.

## Methods

The regional ethical review board in Stockholm, Sweden, approved this study (approval numbers 2015/1137–31/4 and 2018/751–32). The study was performed at the Karolinska University Hospital, Stockholm, Sweden. The hospital is the regional trauma centre for the metropolitan Stockholm area covering more than two million inhabitants. The study adhered to the Strengthening the Reporting of Observational Studies in Epidemiology (STROBE) recommendation for cohort studies [8].

### Patient selection

All individuals, age ≥ 15 years, admitted to the Karolinska University Hospital with full trauma team activation are included in a trauma register. The criteria for trauma team activation has been described previously [9]. In addition, injured individuals admitted to the emergency unit without full trauma team activation but retrospectively found to have an Injury Severity Score (ISS) of 9 or more are also included. Patients with isolated fractures of the upper- or lower extremity, drowning, hypothermia or burns without other traumatic injuries or chronic subdural hematomas are not included. The Abbreviated Injury Scale (AIS) 1990 edition (for 2006) and 2005 edition (from 2007) have been used accordingly. All data including type of injury are recorded according to the Utstein Template [10].

Patients admitted from 1st of January 2006 to 31st of December 2015 were included in the present study; all patients were followed for at least 1 year. Individuals without a valid Swedish identity number, i.e. non-Swedish citizens and immigrants without Swedish citizenship, could not be matched to national register data and were thus excluded. Patients who died within the first 24 h after arrival in hospital without being admitted to the ICU were omitted. These individuals were dead on arrival, died in the trauma unit or the operating theatre, or ICU care was considered futile for other reasons.

### National registers and definitions

The Swedish personal identity number system facilitates linkage of patient data between regional and national registers. Data on comorbidity was gathered from the National In- and Outpatient Registers managed by the Swedish Board of Health and Welfare (NBHW). The registers contain information on all inpatient and outpatient care in Sweden that is not classified as primary care. Diagnoses according to the International Classification of Diseases version 10 (ICD-10) are mandatory. Comorbidity was assessed up to 8 years prior to trauma. Somatic comorbidity, i.e. Charlson Comorbity Index (CCI), were coded from ICD-10 according to the algorithm suggested by Quan et al. [11] Psychiatric comorbidity was defined as the presence of a diagnose in ICD-10 groups F20-F99 and substance abuse as a diagnose in F10-F19 respectively.

Data on anticoagulant use were extracted from the Prescribed Drug Register (NBHW). Use of warfarin or direct oral anticoagulants were defined as having filled at least one prescription with Anatomical Therapeutic Chemical Classification (ATC) codes B01AA03 (warfarin), B01AE07 (dabigatran), B01AF01 (rivaroxaban), B01AF02 (apixaban) or B01AF03 (edoxaban) in the 180 days preceding trauma.

Pre-hospital airway management was defined as oro-tracheal intubation or placement of a laryngeal mask airway prior to arrival in the trauma bay. Nocturnal admission was defined as an admission time between 8.00 pm and 8.00 am. Shock on arrival was defined as a first recorded systolic blood pressure (SAP) < 90 mmHg in the trauma unit. Severe head injury was defined as an injury to the head region with AIS > 2. Mortality at 30-days and 1-year were verified from the Cause of Death Register managed by NBHW. There was no loss to follow up.

### Statistical analysis

Data are presented as numbers and proportions or median with interquartile ranges (IQR) as depicted in the tables. Categorical data were compared with the χ^2^ test and continuous data with the Mann-Whitney U test. The association between gender and ICU-admission were explored using logistic regression analysis. Factors assumed to be associated with ICU-admission were tested in univariate logistic regression; included variables were age (categorised in ten-year intervals), somatic comorbidity (categorised as CCI 0, 1 or > 1 respectively), nocturnal admission, penetrating injury, Injury Severity Score (ISS) (categorised as 0–15, 16–24, 25–40 and > 40 respectively), pre-hospital airway management and shock on arrival. First, the crude association between gender and ICU-admission was analysed. Secondly, a restricted model was constructed adjusting for age and ISS. In the final, comprehensive model, all variables with *p* <  0.10 in univariate analysis were included. The logistic regression was also performed among individuals with or without severe injuries separately, using ISS > 15 as a cut-off. Data were analysed as complete cases, results are presented as odds ratios (OR) with corresponding 95% confidence intervals (CI). Model discrimination was tested by calculating the area under the receiver operating characteristic curve (AUC).

The association between gender and short- and long-term mortality were explored using Cox regression models. Variables known or suspected to be associated with mortality were examined with univariate Cox regression. Candidate variables were age (categorised in ten-year intervals), somatic comorbidity (categorised as CCI 0, 1 or > 1 respectively), psychiatric comorbidity, substance abuse, ISS (categorised as 0–15, 16–24, 25–40 and > 40 respectively), severe head injury, type of injury (penetrating vs. blunt) and shock on arrival. Variables with *p* <  0.10 in univariate analysis were included in the multivariable model. Short-term mortality was assessed at 30-days, long-term mortality at 1 year. Results are presented as hazard ratios (HR) with 95% CI.

A *p*-value < 0.05 was considered statistically significant, all tests two-tailed. Data were analysed using Stata SE 14.2 (StataCorp, College Station, Texas, USA).

## Results

**Fig. 1:**
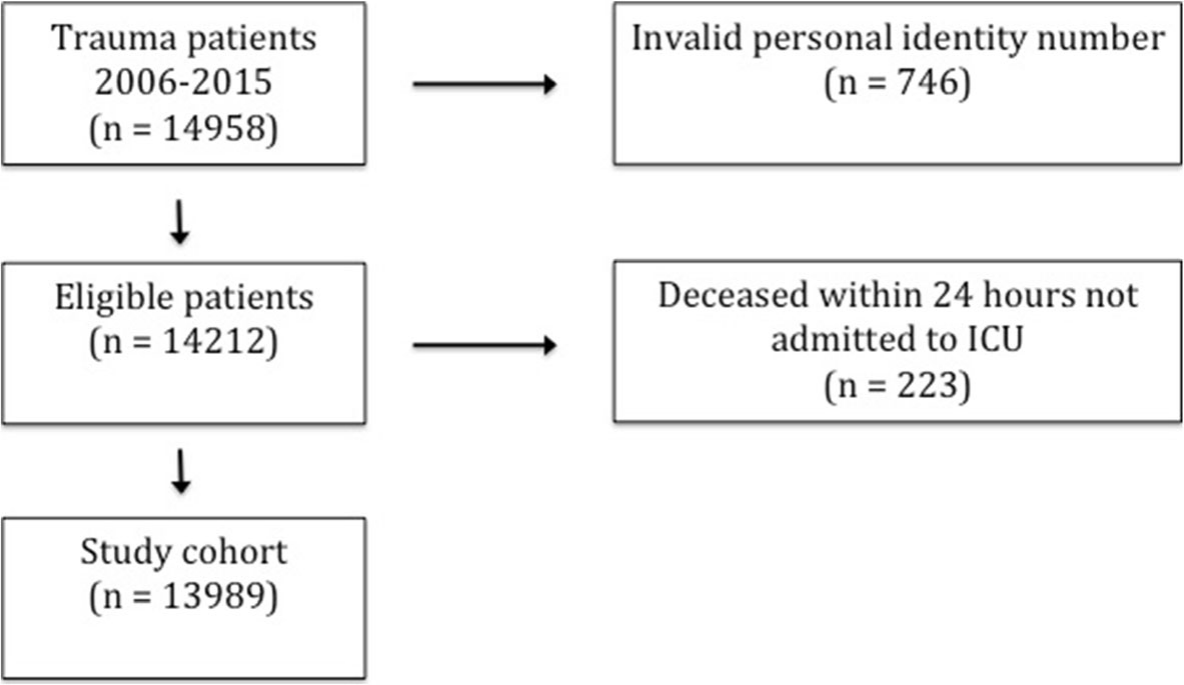
Flow chart of patient inclusion.

Inclusion criteria were met in 13,989 trauma patients (Fig. 1). One third of the included patients were female. Females were older and presented with somatic comorbidity to a greater extent than males, the absolute differences however being small. Traffic related injuries were the most common mechanism of injury in both genders. More males were shot or stabbed and more females presented after low- and high-energy falls. There was no difference between men and women in the proportions presenting with shock on arrival whereas severe injuries were more common among males. More males were admitted to the ICU, (22.1% vs. 15.3%), but short and long-term mortality did not differ between the two groups (Table 1). Among individuals not admitted to the ICU, the differences between men and women were identical to those in the full study cohort (data not shown).

**Table 1:** Baseline characteristics and clinical outcome in the study cohort stratified by gender

	Female	Male	*p*-value
Count (%)	4488 (32.0)	9501 (67.9)	
Age, median (IQR)	40 (24–60)	39 (24–55)	< 0.001
CCI categories, count (%)			< 0.001
0	3500 (78.0)	7676 (80.8)	
1	515 (11.5)	951 (10.0)	
> 1	473 (10.5)	874 (9.2)	
Anticoagulation therapy, count (%)	101 (2.3)	210 (2.2)	0.880
Psychiatric comorbidity, count (%)	884 (19.7)	1546 (16.3)	< 0.001
Substance abuse, count (%)	440 (9.8)	1690 (17.8)	< 0.001
Penetrating injury, count (%)	102 (2.3)	936 (9.9)	< 0.001
Injury mechanism, count (%)			< 0.001
Traffic: motor vehicle	1252 (27.9)	2180 (22.9)	
Traffic: motorcycle	173 (3.9)	1122 (11.8)	
Traffic: bicycle	364 (8.1)	606 (6.4)	
Traffic: pedestrian	323 (7.2)	297 (3.1)	
Traffic: other	29 (0.6)	104 (1.1)	
Shot	11 (0.2)	148 (1.6)	
Stabbed	95 (2.1)	771 (8.1)	
Hit by blunt object	217 (4.8)	1043 (11.0)	
Low-energy fall	712 (15.9)	985 (10.4)	
High-energy fall	1262 (28.1)	2092 (22.0)	
Other	15 (0.3)	83 (0.9)	
Unknown	35 (0.8)	70 (0.7)	
Nocturnal admission, count (%)	1389 (30.9)	3962 (41.7)	< 0.001
ISS, median (IQR)	5 (1–10)	5 (2–14)	< 0.001
ISS, categories			< 0.001
0–15	3771 (84.0)	7475 (78.7)	
16–24	367 (8.2)	1064 (11.2)	
25–40	279 (6.2)	774 (8.1)	
> 40	71 (1.6)	188 (2.0)	
Pre-hospital airway, count (%)	73 (1.6)	201 (2.1)	0.051
Severe head injury, count (%)	619 (13.8)	1557 (16.4)	< 0.001
GCS on arrival, median (IQR)	15 (15–15)	15 (14–15)	< 0.001
GCS on arrival, categories			< 0.001
13–15	3964 (89.2)	8209 (87.0)	
9–12	192 (4.3)	437 (4.6)	
3–8	289 (6.5)	790 (8.4)	
Shock on arrival, count (%)	97 (2.2)	207 (2.2)	0.947
Admitted to ICU, count (%)	687 (15.3)	2099 (22.1)	< 0.001
30-day mortality, count (%)	148 (3.3)	265 (2.8)	0.097
1-year mortality, count (%)	242 (5.4)	465 (4.9)	0.209

Continuous parameters presented as median with interquartile range (IQR), categorical parameters as n (%). *CCI* Charlson Comorbidity Index, *ISS* Injury Severity Score, *GCS* Glasgow Coma Scale

Males were more likely to be admitted to the ICU than females, OR 1.56 (95% CI 1.43–1.72, *p* <  0.001) in univariate logistic regression. This association remained significant after adjustment for potential confounders. In a subgroup analysis the difference was statistically significant only in patients with minor injuries, e.g. ISS ≤ 15 (Table 2, Additional file 1: Table S1). The full model showed excellent discrimination, AUC 0.85.

**Table 2:** Associations between male gender and ICU admission, unadjusted and adjusted OR (95% CI)

	OR (95% CI)	p-value
Unadjusted	1.57 (1.43–1.72)	< 0.001
Restricted model^a^	1.45 (1.29–1.63)	< 0.001
Full model^b^, all patients	1.35 (1.19–1.53)	< 0.001
ISS ≤ 15	1.68 (1.43–1.98)	< 0.001
ISS > 15	0.92 (0.75–1.13)	0.445

*ICU* Intensive Care Unit, *OR* odds ratio, *CI* confidence interval, *ISS* Injury Severity Score

^a^Restricted model: adjusted for age and injury severity

^b^Full model: in addition to the restricted model adjusted for somatic comorbidity, pre-hospital airway, penetrating injury, nocturnal admission and shock on arrival

Among those admitted to ICU after minor injuries, e.g. ISS ≤ 15, men were more often admitted during the night and were more likely to present with penetrating injuries. Women had fall as a trauma mechanism to a greater extent and were in shock on arrival significantly more often. No other significant differences were noted between the two groups (Table 3).

**Table 3:** Baseline characteristics and clinical outcome for individuals admitted to the ICU after minor injuries (ISS ≤ 15), stratified by gender

	Female	Male	*p*-value
Count (%)	220 (22.4)	761 (77.6)	
Age, median (IQR)	44 (21–60)	40 (25–58)	0.591
CCI categories, count (%)			0.672
0	175 (79.5)	585 (76.9)	
1	26 (11.8)	97 (12.7)	
> 1	19 (8.6)	79 (10.4)	
Anticoagulation therapy	7 (3.2)	24 (3.2)	0.983
Psychiatric comorbidity, count (%)	50 (22.7)	147 (19.3)	0.266
Substance abuse, count (%)	45 (20.5)	188 (24.7)	0.192
Penetrating injury, count (%)	19 (8.6)	126 (16.6)	0.004
Injury mechanism, count (%)			< 0.001
Traffic: motor vehicle	26 (11.8)	124 (16.3)	
Traffic: motorcycle	5 (2.3)	84 (11.0)	
Traffic: bicycle	14 (6.4)	12 (1.6)	
Traffic: pedestrian	16 (7.3)	12 (1.6)	
Traffic: other	2 (0.9)	9 (1.2)	
Shot	1 (0.5)	20 (2.6)	
Stabbed	18 (8.2)	98 (12.9)	
Hit by blunt object	19 (8.6)	99 (13.0)	
Low-energy fall	34 (15.5)	90 (11.8)	
High-energy fall	81 (36.8)	173 (22.7)	
Other	2 (0.9)	12 (1.6)	
Unknown	2 (0.9)	5 (0.7)	
Nocturnal admission, count (%)	83 (37.7)	382 (50.2)	< 0.001
ISS, median (IQR)	9 (5–10)	9 (5–11)	0.202
Pre-hospital airway, count (%)	11 (5.0)	35 (4.6)	0.804
Severe head injury, count (%)	48 (21.8)	145 (19.1)	0.364
GCS on arrival, median (IQR)	14 (10–15)	14 (10–15)	0.565
GCS on arrival, categories			0.828
13–15	150 (68.2)	502 (66.0)	
9–12	26 (11.8)	97 (12.7)	
3–8	44 (20.0)	162 (21.3)	
Shock on arrival, count (%)	12 (5.5)	20 (2.6)	0.038
Ventilator days, median (IQR)	0 (0–1)	0 (0–1)	0.496
Hosp. length of stay, days, median (IQR)	5 (2–11)	4 (2–11)	0.429
30-day mortality, count (%)	8 (3.6)	21 (2.8)	0.499
1-year mortality, count (%)	15 (6.8)	41 (5.4)	0.421

Continuous parameters presented as median with interquartile range (IQR) categorical parameters as n (%). *ICU* intensive care unit, *CCI* Charlson Comorbidity Index, *ISS* Injury Severity Score, *GCS* Glasgow Coma Scale

There were no significant differences in crude (HR 0.84, 95% CI 0.69–1.03, *p* = 0.098), or adjusted (HR 1.07, 95% CI 0.86–1.31, *p* = 0.555) 30-day mortality between men and women (Additional file 2: Table S2). At 1 year, men had a significantly increased mortality, HR 1.22 (95% CI 1.03–1.43, *p* = 0.019) after adjustment for age, somatic comorbidity, drug abuse, injury severity, severe head injury, type of injury and shock on arrival (Additional file 3: Table S3). The same patterns of short- and long-term mortality were noted within the subgroup of patients with ISS < 15 (data not shown).

## Discussion

There is a documented male dominance in ICU admission rates in various studies [1–3], however, it is not fully elucidated whether this difference reflects medical needs or is influenced by other factors. In this study we explored post trauma ICU admittance from a gender perspective. In a study cohort of approximately 14,000 trauma patients we showed that men had a higher probability of being admitted to the ICU after initial trauma resuscitation. This difference between men and women in ICU admission rates did not translate into any survival advantages. No differences were noted in short-term survival, whereas men had a higher probability of long-term mortality. The difference between men and women noted in ICU admittance patterns was limited to patients with less severe injuries (ISS < 15). We could not demonstrate any apparent explanations for this difference other than patient gender as such.

The demographics of the study cohort was much in line with studies from other European and North American trauma centres with approximately one third females, a low rate of penetrating injuries and a dominance of road traffic accidents [12, 13]. The median ISS was low and, as expected, overall mortality rates very low.

That men are by far more exposed to trauma is a well-established fact throughout history. Whether patient gender may have an impact on prognosis after severe injury is another question that has been studied both in experimental and clinical settings. In experimental trauma female gender seems to be protective. These findings have been attributed to humoral differences [14]. In clinical studies the pattern is much more diverse. Even though male gender has been shown to be a risk factor in several reports, other investigators have been unable to show this relationship [15, 16]. Thus, it is far from established that patient gender has a significant influence on outcomes in trauma. Gender bias in health care provision is a debated and studied topic. There are data indicating that women receive less aggressive interventional therapy in conjunction with myocardial infarction, possibly linked to worse prognosis [17–19]. In the trauma setting a South-American study showed that, after adjustment for potential confounders, women were less likely to be admitted to a trauma centre [20]. In the field of intensive care, it seems that men are admitted to the ICU to a larger extent [1–3, 21]. Whether this reflects men being more critically ill or if there is, in fact, an existing gender bias is debated. An Austrian multi-centre study comprising over 25,000 ICU patients showed that men received an overall increased level of care despite women having a significantly higher severity of illness on admission. No difference was seen in risk adjusted mortality [3].

We found no apparent explanation for relative overtriage of men to the ICU among patients with ISS ≤ 15. As a consequence, one may expect that undertriage of females to the ICU would generate an increase in severity of illness among admitted women. The demographics of the ICU admitted patients with ISS ≤ 15 reveals very few differences between genders although females had a higher incidence of shock on arrival in support of this assumption. Men and women had different patterns of mechanisms of injury, road traffic accidents and penetrating injuries were more common in men whereas high energy falls more common in women. There were no differences in median ISS or mortality. Clearly all variables taken into account by the attending physician, when considering a patient for ICU admittance, cannot be adjusted for in a statistical analysis. One factor is the perceived severity of illness, this entity may include potential intoxication. We have no record of this in our database apart from the fact that a history of substance abuse was more common and a slightly lower admission GCS was noted for men in the total cohort. Penetrating injuries were seen more often in men potentially influencing liberal ICU admission. Nocturnal time admission was more common in men and may increase the likelihood of ICU admission for the sole purpose of observation, due to reduced staffing in other parts of the hospital during off-hours.

The difference between men and women noted in the current study remained also after adjustment for relevant confounders suggesting that gender bias may actually have influenced the ICU admittance pattern. It cannot be ruled out that this finding reflects a bias among physicians. Interestingly, this aspect was recently studied in survey covering more than 1400 physicians. The study did not reveal any significant gender bias in attitude to ICU admission among Swedish doctors [22]. Nonetheless, our findings suggest a potential difference in attitude based on gender. If this reflects a bias or other factors linked to gender, such as perceived illness cannot be fully deduced in our study.

The gender differences noted in the current study did not generate a significant difference in mortality. However, in the unadjusted Cox regression analysis of the total cohort, there was a tendency to an increased risk of short-term mortality for women. This was not seen in the adjusted analysis. The adjusted hazard ratio for long-term death was increased for men. The latter finding may have several explanations. In a previous study on long-term outcomes after trauma an excessive mortality was noted for several years as compared to a matched cohort [23]. This excessive death was largely explained by trauma recidivism and other external causes of death, a pattern expected to be more common in men than women.

Possible limitations with the current study are associated with the register-based design. Although rather well accounted for, residual confounding can never completely be ruled out. The study is based on patient data from a single centre, but an obvious strength is the large cohort with more than 14,000 patients included. Another strength is the use of well-validated national health registries.

## Conclusion

In this study we found a difference between men and women in post trauma ICU admittance patterns, restricted to less injured patients, where men had a higher probability of ICU admittance. This difference was significant also after adjustment for relevant confounders and cannot be fully explained. Whether this is a true gender bias or an effect of differences in perceived severity of illness or other factors not analysed in this study remains unknown. Clearly this finding warrants further studies.

## Supplementary information

**Additional file 1: Table S1.** Associations between patient- and injury characteristics and ICU admission, adjusted OR (95% CI).

**Additional file 2: Table S2.** Associations between baseline and injury characteristics and 30-day mortality, unadjusted and adjusted HR (95% CI).

**Additional file 3: Table S3.** Associations between baseline and injury characteristics and 1-year mortality, unadjusted and adjusted HR (95% CI).

## Data Availability

The data that support the findings of this study are available from Trauma Registry Karolinska and the National Board of Health and Welfare (NBHW) but restrictions apply to the availability of these data, which were used under license for the current study, and so are not publicly available. Data are however available from the authors upon reasonable request and with permission of Trauma Registry Karolinska and the National Board of Health and Welfare (NBHW).

## Abbreviations

AIS: The Abbreviated Injury Scale
ATC: Anatomical Therapeutic Chemical Classification
AUC: Area Under the Curve
CCI: Charlson Co-morbity Index
CI: Confidence Interval
GCS: Glasgow Coma Scale
HR: Hazard Ratio
ICD 10: International Classification of Diseases version 10
ICU: Intensive care units
IQR: Inter Quartile Range
ISS: Injury Severity Score
NBWH: Swedish National Board of Health and Welfare
OR: Odds Ratio
