# Supplementary material for: Impact of gender on post- traumatic intensive care and outcomes
Source: Scand J Trauma Resusc Emerg Med. 2019 Dec 23;27:115. doi: 10.1186/s13049-019-0693-4 (PMC6929423; doi:10.1186/s13049-019-0693-4)
Supplement: Supplementary file 1 — Additional file 1: Table S1. Associations between patient- and injury characteristics and ICU admission, adjusted OR (95% CI). [file 13049_2019_693_MOESM1_ESM.docx]

| **Supplementary table 1. Associations between patient- and injury characteristics and ICU admission, adjusted OR (95 % CI).** | | | |
| --- | --- | --- | --- |
|  | **OR (95 % CI)** | **p-value** |  |
| **Gender**  **Female**  **Male** | Ref.  1.35 (1.19-1.53) | < 0.001 |  |
| **Age**  **< 30 years**  **30-39 years**  **40-49 years**  **50-59 years**  **60-69 years**  **70-79 years**  **80-89 years**  **> 89 years** | Ref.  0.85 (0.72-1.01)  1.06 (0.90-1.25)  0.98 (0.82-1.17)  1.29 (1.07-1.56)  1.29 (1.02-1.64)  0.81 (0.60-1.09)  0.29 (0.16-0.54) | 0.071  0.482  0.799  0.007  0.034  0.165  < 0.001 |  |
| **CCI, categories**  **0**  **1**  **≥ 2** | Ref.  1.18 (0.99-1.40)  1.08 (0.89-1.31) | 0.066  0.435 |  |
| **Penetrating injury** | 1.77 (1.47-2.12) | < 0.001 |  |
| **Nocturnal admission** | 1.43 (1.28-1.60) | < 0.001 |  |
| **ISS, categories**  **0-15**  **16-24**  **25-40**  **> 40** | Ref.  10.16 (8.93-11.58)  35.66 (30.09-42.27)  284.10 (125.02-645.59) | < 0.001  < 0.001  < 0.001 |  |
| **Prehospital airway** | 247.41 (60.12-1018.21) | < 0.001 |  |
| **Shock on arrival** | 4.22 (2.90-6.13) | < 0.001 |  |

ICU, Intensive Care Unit; OR, odds ratio; CI, confidence interval; CCI, Charlson Comorbidity Index; ISS, Injury Severity Score.
