# Supplementary material for: Impact of gender on post- traumatic intensive care and outcomes
Source: Scand J Trauma Resusc Emerg Med. 2019 Dec 23;27:115. doi: 10.1186/s13049-019-0693-4 (PMC6929423; doi:10.1186/s13049-019-0693-4)
Supplement: Supplementary file 2 — Additional file 2: Table S2. Associations between baseline and injury characteristics and 30-day mortality, unadjusted and adjusted HR (95% CI). [file 13049_2019_693_MOESM2_ESM.docx]

| **Supplementary table 2. Associations between baseline and injury characteristics and 30-day mortality, unadjusted and adjusted HR (95 % CI).** | | | | |
| --- | --- | --- | --- | --- |
|  | **Univariate** | | **Multivariable** | |
|  | **HR (95 % CI)** | **p-value** | **HR (95 % CI)** | **p-value** |
| **Gender**  **Female**  **Male** | Ref.  0.84 (0.69-1.03) | 0.098 | Ref.  1.07 (0.86-1.31) | 0.555 |
| **Age, categories**  **< 30**  **30-39**  **40-49**  **50-59**  **60-69**  **70-79**  **80-89**  **≥ 90** | Ref.  1.03 (0.58-1.82)  1.81 (1.11-2.94)  2.70 (1.71-4.28)  5.87 (3.90-8.81)  13.50 (9.10-20.04)  28.18 (19.44-40.88)  33.60 (21.80-51.81) | 0.928  0.017  < 0.001  < 0.001  < 0.001  < 0.001  < 0.001 | Ref.  1.01 (0.57-1.80)  1.54 (0.95-2.50)  1.81 (1.14-2.88)  3.23 (2.12-4.94)  6.73 (4.42-10.27)  18.26 (12.17-27.41)  31.69 (19.61-51.20) | 0.965  0.083  0.012  < 0.001  < 0.001  < 0.001  < 0.001 |
| **CCI, categories**  **0**  **1**  **≥ 2** | Ref.  2.76 (2.10-3.62)  6.43 (5.19-7.97) | < 0.001  < 0.001 | Ref.  0.94 (0.71-1.26)  1.77 (1.38-2.25) | 0.700  < 0.001 |
| **Psychiatric comorbidity** | 0.95 (0.74-1.23) | 0.718 |  |  |
| **Substance abuse** | 0.87 (0.66-1.15) | 0.338 |  |  |
| **ISS, categories**  **0-15**  **16-24**  **25-40**  **> 40** | Ref.  5.52 (4.02-7.57)  24.61 (19.24-31.47)  30.85 (22.24-42.81) | < 0.001  < 0.001  < 0.001 | Ref.  2.63 (1.88-3.69)  10.32 (7.68-13.86)  20.36 (13.71-30.23) | < 0.001  < 0.001  < 0.001 |
| **Severe head injury** | 12.91 (10.48-15.91) | < 0.001 | 2.86 (2.22-3.68) | < 0.001 |
| **Penetrating injury** | 0.57 (0.35-0.91) | 0.018 | 1.97 (1.20-3.22) | 0.007 |
| **Shock on arrival** | 7.86 (5.93-10.42) | < 0.001 | 2.23 (1.63-3.06) | < 0.001 |

HR, hazard ratio; CI, confidence interval; CCI, Charlson Comorbidity Index; ISS, Injury Severity Score.
