# Supplementary material for: Impact of gender on post- traumatic intensive care and outcomes
Source: Scand J Trauma Resusc Emerg Med. 2019 Dec 23;27:115. doi: 10.1186/s13049-019-0693-4 (PMC6929423; doi:10.1186/s13049-019-0693-4)
Supplement: Supplementary file 3 — Additional file 3: Table S3. Associations between baseline and injury characteristics and 1-year mortality, unadjusted and adjusted HR (95% CI). [file 13049_2019_693_MOESM3_ESM.docx]

| **Supplementary table 3. Associations between baseline and injury characteristics and 1-year mortality, unadjusted and adjusted HR (95 % CI).** | | | | |
| --- | --- | --- | --- | --- |
|  | **Univariate** | | **Multivariable** | |
|  | **HR (95 % CI)** | **p-value** | **HR (95 % CI)** | **p-value** |
| **Gender**  **Female**  **Male** | Ref.  0.90 (0.77-1.06) | 0.205 | Ref.  1.22 (1.03-1.43) | 0.019 |
| **Age, categories**  **< 30**  **30-39**  **40-49**  **50-59**  **60-69**  **70-79**  **80-89**  **≥ 90** | Ref.  0.97 (0.60-1.57)  1.89 (1.26-2.80)  3.46 (2.42-4.94)  7.38 (5.36-10.19)  17.04 (12.45-23.33)  32.41 (23.94-43.89)  53.34 (38.20-74.47) | 0.917  0.002  < 0.001  < 0.001  < 0.001  < 0.001  < 0.001 | Ref.  0.92 (0.57-1.49)  1.60 (1.08-2.38)  2.18 (1.52-3.14)  4.16 (2.98-5.81)  9.15 (6.54-12.81)  22.79 (16.35-31.77)  49.28 (33.98-71.47) | 0.732  0.020  < 0.001  < 0.001  < 0.001  < 0.001  < 0.001 |
| **CCI, categories**  **0**  **1**  **≥ 2** | Ref.  3.33 (2.70-4.10)  8.66 (7.36-10.19) | < 0.001  < 0.001 | Ref.  1.22 (0.98-1.51)  2.37 (1.97-2.84) | 0.081  < 0.001 |
| **Psychiatric comorbidity** | 1.14 (0.95-1.38) | 0.160 |  |  |
| **Substance abuse** | 1.24 (1.03-1.50) | 0.026 | 1.59 (1.29-1.96) | < 0.001 |
| **ISS, categories**  **0-15**  **16-24**  **25-40**  **> 40** | Ref.  3.10 (2.49-3.86)  10.23 (8.62-12.14)  12.17 (9.33-15.88) | < 0.001  < 0.001  < 0.001 | Ref.  1.68 (1.33-2.13)  5.20 (4.20-6.44)  10.88 (7.89-14.99) | < 0.001  < 0.001  < 0.001 |
| **Severe head injury** | 7.50 (6.46-8.69) | < 0.001 | 2.29 (1.91-2.76) | < 0.001 |
| **Penetrating injury** | 0.55 (0.38-0.79) | 0.001 | 1.49 (1.02-2.17) | 0.039 |
| **Shock on arrival** | 5.53 (4.31-7.10) | < 0.001 | 2.08 (1.57-2.74) | < 0.001 |

HR, hazard ratio; CI, confidence interval; CCI, Charlson Comorbidity Index; ISS, Injury Severity Score.
